# N-Terminal Pro-brain Natriuretic Peptide Is Associated With Hemorrhagic Transformation and Poor Outcomes in Patients With Stroke Treated With Intravenous Thrombolysis

**DOI:** 10.3389/fnmol.2021.758915

**Published:** 2021-11-22

**Authors:** Ke-Jia Zhang, Hang Jin, Rui Xu, Peng Zhang, Zhen-Ni Guo, Yi Yang

**Affiliations:** ^1^Department of Neurology, China National Comprehensive Stroke Center, The First Hospital of Jilin University, Changchun, China; ^2^Department of Neurology, Clinical Trial and Research Center for Stroke, The First Hospital of Jilin University, Changchun, China

**Keywords:** stroke, NT-proBNP, mortality, functional outcome, hemorrhagic transformation

## Abstract

**Background:** N-terminal pro-brain natriuretic peptide (NT-proBNP) levels are a promising biomarker for predicting stroke outcomes; however, their prognostic validity is not well-understood in patients who have undergone intravenous thrombolysis. This study was designed to evaluate the prognostic value of NT-proBNP levels in patients with acute ischemic stroke treated with intravenous thrombolysis.

**Methods:** Patients with ischemic stroke who underwent intravenous thrombolysis between April 2015 and December 2020 were analyzed. Demographic information, information related to intravenous thrombolysis, medical history, and laboratory test results were collected. Outcomes, such as hemorrhagic transformation, early neurologic deterioration, poor 3-month functional outcomes, and 3-month mortality were recorded. Correlations between NT-proBNP levels and the above outcomes were analyzed, an individualized prediction model based on NT-proBNP levels for functional outcomes was developed, and a nomogram was drafted.

**Results:** A total of 404 patients were included in the study. Elevated NT-proBNP levels were independently associated with hemorrhagic transformation, poor 3-month functional outcomes, and 3-month mortality, while early neurological deterioration was not. An association between NT-proBNP levels and hemorrhagic transformation was noted. An individualized prediction model for poor functional outcomes was established, which was composed of ln(NT-proBNP), National Institutes of Health Stroke Scale (NIHSS), and baseline glucose, with good discrimination [area under the curve (AUC) 0.764] and calibration (*P* > 0.05).

**Conclusion:** To the best of our knowledge, this is the first report on the association between NT-proBNP levels and hemorrhagic transformation in patients who have undergone intravenous thrombolysis. The 3-month functional outcomes and mortality were found to be associated with NT-proBNP levels. An individualized prediction model based on NT-proBNP levels to predict the 3-month functional outcomes was established. Our results suggest that NT-proBNP levels could be used as a prognostic biomarker in patients with acute ischemic stroke treated with intravenous thrombolysis.

## Introduction

N-terminal pro-brain natriuretic peptide (NT-proBNP) is an inactive fragment derived from the cleavage of BNP, which is released by the ventricular myocardium in response to stretching (Daniels and Maisel, 2007). NT-proBNP is associated with cardioembolic stroke, stroke-related atrial fibrillation (AF), and atrial cardiopathy (Llombart et al., 2015; Kamel et al., 2018; Zhang K. et al., 2021) and is also associated with poor outcomes in patients with stroke. Previous studies have reported that NT-proBNP levels are associated with mortality, poor functional outcomes, and stroke-associated complications (Garcia-Berrocoso et al., 2013; Zhao et al., 2020; Faura et al., 2021).

Intravenous thrombolysis is one of the few approved treatments for patients with acute ischemic stroke, and it can greatly improve survival and functional status (Muruet et al., 2018). Most studies did not differentiate patients who underwent intravenous thrombolysis (Garcia-Berrocoso et al., 2013; Zhao et al., 2020; Faura et al., 2021), and few have reported the prognostic value of NT-proBNP levels in patients with acute ischemic stroke treated with intravenous thrombolysis. One study reported a higher modified Rankin scale (mRS) in patients with high BNP levels who underwent intravenous thrombolysis (Gupta et al., 2019). Similarly, another study reported that increased NT-proBNP levels were associated with malignant edema and mortality in patients who had undergone intravenous thrombolysis or endovascular treatment (Zhang X. et al., 2021). The prognostic validity of NT-proBNP levels in patients with stroke who underwent intravenous thrombolysis is not as well-understood. In this study, we aimed to further investigate the prognostic value of NT-proBNP levels in patients with acute ischemic stroke who received intravenous thrombolysis and to evaluate its association with mortality, functional outcomes, hemorrhagic transformation, and early neurological deterioration.

## Materials and Methods

### Patients

A retrospective analysis of a prospectively collected database of consecutive patients with acute ischemic stroke who underwent intravenous thrombolysis from April 2015 to December 2020 was performed. The diagnosis of acute ischemic stroke was based on baseline symptoms and CT imaging performed before intravenous thrombolysis, which was further confirmed by follow-up CT or MRI. All patients who underwent intravenous thrombolysis met the inclusion criteria for intravenous thrombolysis according to the 2018 American Heart Association/American Stroke Association (AHA/ASA) guidelines (Furie and Jayaraman, 2018). Recombinant tissue plasminogen activator (rt-PA) was infused at the emergency department within 4.5 h of stroke onset; a dosage of 0.6 or 0.9 mg/kg was used. Blood samples were drawn within 24 h of intravenous thrombolysis administration and sent to the clinical laboratory for immediate measurement, and NT-proBNP levels were measured within 24 h after intravenous thrombolysis administration. A second CT scan was performed 24 h after intravenous thrombolysis administration to determine hemorrhagic transformation. The subtypes of stroke were categorized with the criteria developed for the Trial of Org 10172 in Acute Stroke Treatment (TOAST) (Adams et al., 1993). Patients with available NT-proBNP results were included in the present study. Patients were excluded from the present study if they (1) had incomplete clinical data or (2) had heart failure, valvular disease, or myocardial infarction.

### Data Collection and Outcomes

Demographic information [age, sex, body mass index (BMI)], information related to intravenous thrombolysis [onset to needle time (ONT), dosage of rt-PA, bridging therapy], lifestyle history (smoking and drinking), medical history [hypertension, diabetes mellitus, AF, and coronary heart disease (CHD)], baseline and 24 h National Institutes of Health Stroke Scale (NIHSS) scores, baseline systolic blood pressure (sBP) and diastolic blood pressure (dBP), baseline fingertip blood glucose, laboratory results [NT-proBNP, low density lipoprotein-cholesterol (LDL-C), high-density lipoprotein-cholesterol (HDL-C), triglyceride, total cholesterol, and homocysteine levels] were collected. Dyslipidemia was defined according to the Chinese guidelines for the prevention and treatment of dyslipidemia (Joint Committee for Developing Chinese guidelines on Prevention and Treatment of Dyslipidemia in Adults, 2007).

Hemorrhagic transformation was assessed on the second CT scan 24 h after intravenous thrombolysis. Early neurological deterioration was defined as an increase in NIHSS score ≥4 at 24 h after thrombolysis. A 3-month follow-up was performed, at which point, the patients’ mortality and functional outcomes were assessed. Functional outcomes were assessed with 3-month mRS, and a good functional outcome was defined as a mRS of 0–2, whereas a poor functional outcome was defined as a mRS of 3–6.

### Statistical Analysis

Continuous variables are expressed as mean and SD, median, and interquartile range (IQR) according to normal distribution. Categorical variables are expressed as percentages. The comparison between continuous variables was tested using the *t*-test or Mann-Whitney *U* test according to normality. Comparisons between categorical variables were assessed using the *X*^2^ test. Binary logistic regression and sensitivity analyses were performed to test the correlation stability, and univariate and multivariate analyses were performed. Univariate analysis was performed to determine factors related to poor outcome in stroke with intravenous thrombolysis. NT-proBNP was performed with natural log transformations, and ln(NT-proBNP) was fitted in. The TOAST criteria were fitted in, which included large artery atherosclerosis, cardioembolism, small artery occlusion lacunar, and undetermined etiology, with cardioembolism as the reference. Because of limited population of stroke with other etiology, only subtypes of stroke were analyzed in univariate analysis. Multivariate analysis was then performed with all included patients. Four models were developed for the multivariate analysis: Model 1 was adjusted for age, sex, BMI, ONT, dosage of rt-PA, bridging therapy, and baseline NIHSS score; Model 2 was adjusted for cardiovascular risk factors, such as drinking, smoking, prior stroke, AF, CHD, hypertension, diabetes mellitus, and dyslipidemia; Model 3 was adjusted for baseline blood pressure and laboratory tests, such as sBP, dBP, blood glucose, total cholesterol, triglyceride, LDL-C, HDL-C, and homocysteine levels; Model 4 was adjusted using Model 1 + Model 2 + Model 3. The association was assessed with *P*-value, odds ratio (OR), and 95% CI. An individualized prediction model based on NT-proBNP for functional outcomes was then performed using multivariable logistic regression analysis. Seventy percent of the cases were regarded as the training group for model development, with 30% as the validation group for testing the model’s performance. A 10-fold cross-validation was also performed in all patients. Random sampling was used to determine the training and validation groups. Backward stepwise selection was applied using the likelihood ratio test with Akaike’s information criterion as the stopping rule. The nomogram was drafted accordingly, based on the data from the training group. The discrimination of the prediction model was assessed using the receiver operating characteristic (ROC) and area under the curve (AUC), and calibration of the prediction model was assessed using the Hosmer-Lemeshow test. Discrimination and calibration were calculated for both the training and validation groups. Statistical analysis was completed using SPSS 21.0, and the nomogram and 10-fold cross-validation were performed with Stata 15.0.

## Results

### Characteristics of Included Patients

**FIGURE 1:**
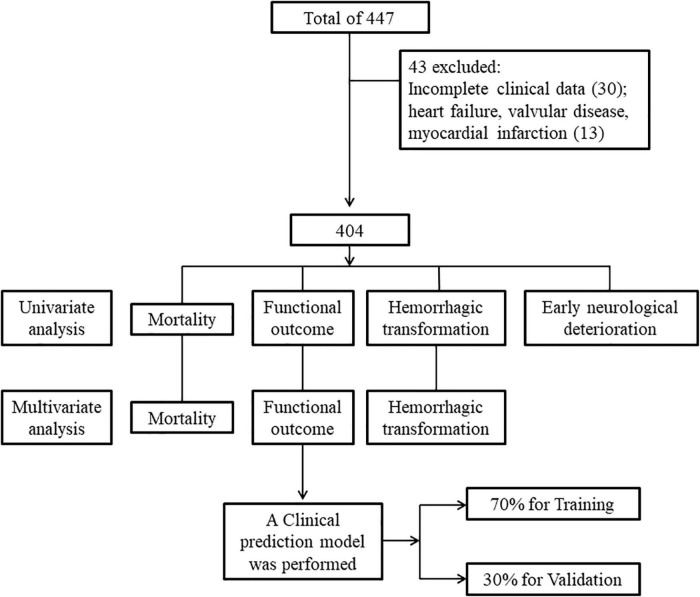
Flowchart of patient selection. Four hundred and four patients were finally included in the study after exclusion. Univariate analysis was performed regarding mortality, functional outcome, hemorrhagic transformation, and early neurologic deterioration as outcome. Multivariate analysis was performed regarding mortality, functional outcome, and hemorrhagic transformation as outcome. A clinical prediction model was then performed with 70% patients as training group and 30% as validation group, regarding functional outcome as the outcome.

**FIGURE 2:**
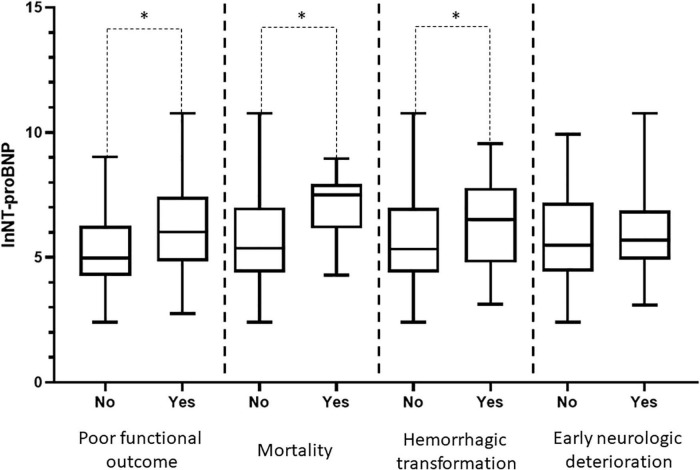
N-terminal pro-brain natriuretic peptide (NT-proBNP) in poor functional outcome, mortality, hemorrhagic transformation, and early neurologic deterioration. One hundred seventy-seven patients had good functional outcomes and 227 had poor functional outcomes; 30 patients died, 374 survived; 72 patients had hemorrhagic transformation, 332 not; 31 patients had early neurological deterioration, 373 not. Distribution of NT-proBNP was depicted with natural log transformations, which was depicted with median and interquartile range. Patients with poor functional outcome, mortality, and hemorrhagic transformation had higher NT-proBNP levels compared with patients without. *P < 0.05.

A total of 447 patients were included in the present study. Thirty patients were excluded because they had incomplete clinical data, including 21 with incomplete laboratory test data and 9 who had data lost during follow-up. Thirteen patients were excluded because of heart failure, valvular disease, or myocardial infarction. A total of 404 patients were included in the final analysis. The flowchart of patient selection is depicted in Figure 1. 71.3% of patients were administered rt-PA 0.9 mg/kg, and 28.7% were administered rt-PA 0.6 mg/kg. In addition, 13.4% of the patients underwent bridging therapy. A total of 177 patients had good functional outcomes, and 227 had poor functional outcomes, compared to mRS 0–1 before stroke onset. Hemorrhagic transformation was observed in 72 patients, early neurological deterioration was observed in 31 patients, and 30 patients died within 3 months. Patients with poor functional outcome, mortality, and hemorrhagic transformation had higher NT-proBNP levels compared with patients without, while patients with early neurologic deterioration not. Distribution of NT-proBNP levels in patients with poor functional outcome, mortality, hemorrhagic transformation, and early neurologic deterioration are shown in Figure 2.

### Comparison of Clinical Characteristics in Patients With Good and Poor Functional Outcomes

Patients with poor outcomes tended to have higher NIHSS scores and higher sBP, glucose, homocysteine, and NT-proBNP levels. NT-proBNP levels in patients with good and poor functional outcomes were 145.00 (70.65–526.50) and 411.00 (128.00–1690.00) pg/ml, respectively. The characteristics of the included patients are shown in Table 1.

**TABLE 1:** Characteristics of included patients.

	Good outcome	Poor outcome	*P*-value
No.	177	227	
Gender (male)	66.7%	70.9%	0.386
Age	63.87 (11.35)	63.87 (11.35)	0.058
BMI	24.02 (22.86–25.25)	24.28 (23.44–25.61)	0.221
ONT	171.02 (54.33)	171.02 (54.33)	0.091
Dosage 0.9 mg/kg	71.8%	70.9%	0.912
Bridging therapy	10.7%	15.4%	0.187
NIHSS	8.00 (5.00–12.00)	12.00 (8.00–15.00)	<0.001
Smoking	46.9%	41.9%	0.315
Drinking	36.2%	41.0%	0.355
AF	10.7%	10.1%	0.871
CHD	22.0%	26.0%	0.413
Hypertension	38.4%	44.5%	0.225
Diabetes	17.5%	20.7%	0.448
Prior Stroke	13.0%	17.6%	0.216
Dyslipidemia	22.6%	26.9%	0.355
sBP	154.45 (18.62)	149.53 (19.48)	0.010
dBP	90 (14.30)	88.3 (13.46)	0.223
Glucose	5.58 (4.84–7.14)	6.33 (5.26–8.30)	<0.001
Homocysteine	14.3 (10.90–20.90)	15.90 (11.60–24.30)	0.041
Cholesterol	4.78 (1.19)	4.78 (0.92)	0.639
Triglyceride	1.36 (0.95–1.94)	1.16 (0.85–1.71)	0.279
LDL-C	2.92 (0.91)	2.9 (0.71)	0.795
HDL-C	1.22 (1.03–1.43)	1.24 (1.04–1.50)	0.194
NT-proBNP	145.00 (70.65–526.50)	411.00 (128.00–1690.00)	<0.001

*Categorical variables were expressed as percentage, %; Continuous variables conform to normal distribution were expressed as mean and SD, Mean (SD); Continuous variables not conform to normal distribution were expressed as median and IQR, Median (Q1–Q3). Gender (male), BMI (kg/m^2^), ONT (min), Homocysteine (μmol/L), Glucose (mmol/L), BP (mmHg), Cholesterol (mmol/L), Triglyceride (mmol/L), LDL-C (mmol/L), HDL-C (mmol/L), NT-proBNP (pg/mL). BMI, Body mass index; ONT, Onset to needle time; NIHSS, The National Institutes of Health Stroke Scale; AF, Atrial fibrillation; CHD, Coronary heart disease; sBP, Systolic blood pressure; dBP, Diastolic blood pressure; LDL-C, Low density lipoprotein cholesterin; HDL-C, High density lipoprotein cholesterin; NT-proBNP, The N-terminal of the prohormone brain natriuretic peptide; SD, Standard deviation; IQR, Interquartile range.*

### Univariate and Multivariate Analysis

In univariate analysis, NT-proBNP levels, NIHSS scores, baseline sBP, baseline glucose, and homocysteine levels were associated with poor functional outcomes. NT-proBNP levels, NIHSS scores, smoking, AF, baseline glucose, homocysteine, and LDL-C levels were associated with mortality. NT-proBNP levels, smoking, drinking, hypertension, and bridging therapy were positively associated with hemorrhagic transformation, while cholesterol and LDL-C levels were negatively associated. NT-proBNP levels were associated with poor functional outcomes, mortality, and hemorrhagic transformation. NT-proBNP levels were not associated with early neurologic deterioration; however, hypertension, diabetes mellitus, and baseline glucose levels were positively associated with early neurologic deterioration. The results of the univariate analysis are depicted in Table 2.

**TABLE 2:** Results of univariate analysis.

	Functional outcome			Mortality			Hemorrhagic transformation			Early neurologic deterioration		
				
	*P*-value	OR	95%CI	*P*-value	OR	95%CI	*P*-value	OR	95%CI	*P*-value	OR	95%CI
Gender	0.359	1.220	0.80–1.86	0.908	1.049	0.47–2.36	0.140	1.560	0.86–2.82	0.521	1.314	0.57–3.02
Age	0.059	1.017	1.00–1.03	0.056	1.035	1.00–1.07	0.925	0.999	0.98–1.02	0.895	0.998	0.97–1.03
BMI	0.221	1.043	0.97–1.12	0.692	0.974	0.86–1.11	0.291	1.048	0.96–1.14	0.566	1.037	0.92–1.17
ONT	0.092	1.003	1.00–1.01	0.155	1.005	1.00–1.01	0.155	1.003	1.00–1.01	0.375	1.003	1.00–1.01
Dosage 0.9 mg/kg	0.855	0.960	0.62–1.48	0.871	0.935	0.41–2.11	0.631	1.151	0.65–2.05	0.967	0.983	0.44–2.20
Bridging therapy	0.172	1.516	0.83–2.75	0.575	0.704	0.21–2.40	0.006	2.464	1.30–4.68	0.123	2.023	0.83–4.95
NIHSS	<0.001	1.124	1.08–1.17	<0.001	1.112	1.07–1.16	0.150	1.025	0.99–1.06	0.076	0.939	0.88–1.01
Smoking	0.311	0.815	0.55–1.21	0.031	2.336	1.08–5.05	0.031	1.757	1.05–2.94	0.804	0.910	0.43–1.91
Drinking	0.325	1.225	0.82–1.84	0.602	1.221	0.58–2.59	0.001	2.463	1.47–4.13	0.434	0.732	0.34–1.60
AF	0.844	0.938	0.49–1.78	0.004	3.636	1.50–8.79	0.058	2.013	0.98–4.15	0.892	0.918	0.27–3.16
CHD	0.358	1.243	0.78–1.97	0.232	1.625	0.73–3.60	0.657	0.871	0.47–1.60	0.834	1.094	0.47–2.53
Hypertension	0.220	1.285	0.86–1.92	0.833	0.921	0.43–1.97	0.010	1.967	1.18–3.29	0.026	2.354	1.11–4.99
Diabetes	0.421	1.230	0.74–2.03	0.920	1.049	0.41–2.66	0.309	1.373	0.75–2.53	0.021	2.512	1.15–5.49
Prior Stroke	0.205	1.432	0.82–2.50	0.385	0.581	0.17–1.98	0.526	1.243	0.63–2.43	0.549	1.331	0.52–3.39
Dyslipidemia	0.326	1.259	0.80–1.99	0.276	1.555	0.70–3.44	1.000	1.000	0.56–1.80	0.334	1.476	0.67–3.25
sBP	0.011	1.014	1.00–1.02	0.598	1.005	0.99–1.03	0.400	1.006	0.99–1.02	0.063	1.020	1.00–1.04
dBP	0.223	1.009	0.99–1.02	0.516	0.991	0.96–1.02	0.658	1.004	0.99–1.02	0.883	0.998	0.97–1.02
Glucose	<0.001	1.157	1.07–1.26	0.015	1.144	1.03–1.27	0.092	1.075	0.99–1.17	0.001	1.194	1.08–1.32
Homocysteine	0.049	1.014	1.00–1.03	0.041	1.019	1.00–1.04	0.239	1.009	0.99–1.02	0.796	0.997	0.97–1.02
Cholesterol	0.977	0.997	0.83–1.20	0.101	1.306	0.95–1.80	0.022	0.741	0.57–0.96	0.786	0.953	0.68–1.34
Triglyceride	0.280	0.890	0.72–1.10	0.477	0.848	0.54–1.34	0.530	0.911	0.68–1.22	0.168	1.262	0.91–1.76
LDL-C	0.753	1.039	0.82–1.32	0.028	1.565	1.05–2.34	0.005	0.606	0.43–0.86	0.910	0.975	0.62–1.52
HDL-C	0.195	1.462	0.82–2.60	0.112	2.125	0.84–5.39	0.503	0.772	0.36–1.65	0.492	0.674	0.22–2.07
ln(NT-proBNP)	<0.001	1.451	1.26–1.67	<0.001	1.788	1.38–2.31	<0.001	1.341	1.14–1.58	0.676	1.051	0.83–1.32

*OR, Odds ratio; 95% CI, 95% confidence interval; BMI, Body mass index; ONT, Onset to needle time; NIHSS, The National Institutes of Health Stroke Scale; AF, Atrial fibrillation; CHD, Coronary heart disease; sBP, Systolic blood pressure; dBP, Diastolic blood pressure; LDL-C, Low density lipoprotein cholesterin; HDL-C, High density lipoprotein cholesterin; NT-proBNP, N-terminal of the prohormone brain natriuretic peptide.*

Considering the subtypes of stroke, 105 patients were categorized as large artery atherosclerosis, 98 were categorized as cardioembolism, 125 were categorized as small artery occlusion lacunar, 3 were categorized as other etiology, and 73 were categorized as undetermined etiology. Stroke with other etiology was eliminated for limited population, and 401 patients were included in the following analysis. With cardioembolism as the reference, stroke with large artery atherosclerosis was negatively associated with hemorrhage transformation. Small artery occlusion was negatively associated with poor functional outcome, mortality, and hemorrhagic transformation. The association between stroke subtypes and stroke outcome is depicted in Table 3. The association between NT-proBNP and stroke outcome in patients who underwent intravenous thrombolysis was further evaluated in subtypes of stroke, which found that NT-proBNP was associated with functional outcome in large artery atherosclerosis, undetermined etiology, and hemorrhagic transformation in large artery atherosclerosis, however, not remarkable in other subtypes. The association between NT-proBNP and stroke outcome in subtypes of stroke are depicted in Table 4.

**TABLE 3:** Association between stroke subtypes and stroke outcome.

Stroke subtype	No. patients	Poor functional outcome			Mortality			Hemorrhagic transformation		
				
		*P*-value	OR	95%CI	*P*-value	OR	95%CI	*P*-value	OR	95%CI
Cardioembolism	98	As the reference								
Large artery atherosclerosis	105	0.125	1.58	0.88–2.83	0.081	0.43	0.17–1.11	0.009	0.39	0.19–0.79
Small artery occlusion lacunar	125	0.004	0.46	0.27–0.78	0.004	0.05	0.01–0.38	0.011	0.42	0.22–0.82
Undetermined etiology	73	0.864	0.95	0.51–1.76	0.522	0.74	0.29–1.87	0.067	0.49	0.23–1.05

*OR, Odds ratio; 95% CI, 95% confidence interval.*

**TABLE 4:** Association between NT-proBNP and stroke outcome in subtypes of stroke.

Subtypes	Functional outcome			Mortality			Hemorrhagic transformation		
			
	*P*-value	OR	95%CI	*P*-value	OR	95%CI	*P*-value	OR	95%CI
Large artery atherosclerosis	0.003	1.89	1.25–2.86	0.077	1.60	0.95–2.69	0.027	1.56	1.05–2.32
Caridoembolism	0.359	1.19	0.82–1.72	0.837	1.23	0.18–8.54	0.436	1.22	0.74–2.02
Small artery occlusion lacunar	0.436	1.22	0.74–2.02	0.156	1.53	0.85–2.76	0.581	1.12	0.74–1.70
Undetermined etiology	0.002	1.71	1.22–2.41	0.096	1.49	0.93–2.38	0.337	1.20	0.83–1.75

*OR, Odds ratio; 95% CI, 95% confidence interval.*

In multivariate analysis, NT-proBNP levels were independently associated with poor functional outcomes, mortality, and hemorrhagic transformation, adjusted by Models 1, 2, 3, and 4. In Model 1, the ORs for poor functional outcomes, mortality, and hemorrhagic transformation were 1.36 (95% CI, 1.16–1.59), 1.68 (95% CI 1.27–2.24), and 1.42 (95% CI 1.18–1.72), respectively. In Model 2, the ORs for poor functional outcomes, mortality, and hemorrhagic transformation were 1.62 (95% CI 1.37–1.91), 1.75 (95% CI 1.31–2.32), and 1.43 (95% CI 1.18–1.74), respectively. In Model 3, the ORs for poor functional outcomes, mortality, and hemorrhagic transformation were 1.39 (95% CI 1.20–1.61), 1.74 (95% CI 1.32–2.30), and 1.32 (95% CI 1.11–1.58), respectively. In Model 4, the ORs for poor functional outcomes, mortality, and hemorrhagic transformation were 1.45 (95% CI 1.20–1.76), 1.59 (95% CI 1.12–2.23), and 1.58 (95% CI 1.26–1.99), respectively. The results of the multivariate analysis are depicted in Table 5. Multivariate analysis for early neurologic deterioration was not performed.

**TABLE 5:** Results of multivariate analysis.

		Functional outcome			Mortality			Hemorrhagic transformation		
			
		*P*-value	OR	95%CI	*P*-value	OR	95%CI	*P*-value	OR	95%CI
Model 1	ln(NT-proBNP)	<0.001	1.36	1.16–1.59	<0.001	1.68	1.27–2.24	<0.001	1.42	1.18–1.72
	Gender	0.170	1.40	0.87–2.26	0.495	1.36	0.56–3.31	0.115	1.65	0.89–3.07
	Age	0.823	1.00	0.98–1.02	0.802	1.01	0.97–1.05	0.474	0.99	0.97–1.02
	BMI	0.100	1.07	0.99–1.15	0.731	1.02	0.89–1.17	0.318	1.05	0.96–1.15
	ONT	0.130	1.00	1.00–1.01	0.503	1.00	0.99–1.01	0.164	1.00	1.00–1.01
	Dosage 0.9 mg/kg	0.539	1.17	0.71–1.92	0.595	1.28	0.52–3.17	0.406	1.30	0.70–2.42
	Bridging therapy	0.681	1.15	0.60–2.20	0.455	0.61	0.17–2.22	0.011	2.41	1.22–4.75
	NIHSS	<0.001	1.10	1.05–1.14	<0.001	1.09	1.05–1.15	0.878	1.00	0.96–1.04
Model 2	ln(NT-proBNP)	<0.001	1.62	1.37–1.91	<0.001	1.75	1.31–2.32	<0.001	1.43	1.18–1.74
	Smoking	0.060	0.63	0.39–1.02	0.045	2.61	1.02–6.63	0.707	1.13	0.60–2.13
	Drinking	0.069	1.57	0.97–2.54	0.732	0.85	0.34–2.14	0.002	2.70	1.43–5.12
	AF	0.014	0.40	0.19–0.83	0.293	1.73	0.62–4.83	0.624	1.23	0.53–2.84
	CHD	0.327	0.77	0.45–1.30	0.915	1.05	0.43–2.54	0.096	0.56	0.28–1.11
	Hypertension	0.381	1.23	0.77–1.96	0.607	0.78	0.31–1.99	0.032	1.92	1.06–3.47
	Diabetes	0.914	1.03	0.58–1.84	0.708	1.24	0.40–3.79	0.499	1.28	0.63–2.58
	Prior stroke	0.513	1.23	0.67–2.25	0.361	0.53	0.14–2.06	0.855	0.93	0.45–1.96
	Dyslipidemia	0.224	1.36	0.83–2.22	0.487	1.37	0.57–3.29	0.492	0.80	0.42–1.52
Model 3	ln(NT-proBNP)	<0.001	1.39	1.20–1.61	<0.001	1.74	1.32–2.30	0.002	1.32	1.11–1.58
	sBP	0.127	1.01	1.00–1.02	0.524	1.01	0.99–1.03	0.577	1.00	0.99–1.02
	dBP	0.753	1.00	0.99–1.02	0.337	0.99	0.96–1.01	0.826	1.00	0.98–1.02
	Glucose	0.006	1.13	1.03–1.23	0.103	1.11	0.98–1.26	0.119	1.08	0.98–1.18
	Homocysteine	0.042	1.02	1.00–1.03	0.028	1.02	1.00–1.04	0.305	1.01	0.99–1.02
	Cholesterol	0.512	0.83	0.47–1.45	0.615	0.77	0.29–2.10	0.277	1.49	0.73–3.04
	Triglyceride	0.855	1.03	0.76–1.39	0.583	0.83	0.43–1.61	0.901	0.98	0.67–1.43
	LDL-C	0.507	1.24	0.66–2.33	0.203	2.01	0.69–5.86	0.030	0.40	0.18–0.91
	HDL-C	0.220	1.67	0.74–3.78	0.340	2.05	0.47–8.92	0.371	0.63	0.22–1.75
Model 4	ln(NT-proBNP)	<0.001	1.45	1.20–1.76	0.009	1.59	1.12–2.23	0.000	1.58	1.26–1.99
	Gender	0.182	1.49	0.83–2.68	0.928	0.95	0.29–3.11	0.836	0.92	0.41–2.04
	Age	0.929	1.00	0.98–1.03	0.532	1.02	0.97–1.06	0.731	0.99	0.97–1.02
	BMI	0.266	1.05	0.97–1.14	0.775	1.02	0.88–1.19	0.067	1.11	0.99–1.23
	ONT	0.188	1.00	1.00–1.01	0.349	1.00	1.00–1.01	0.114	1.00	1.00–1.01
	Dosage 0.9 mg/kg	0.550	1.17	0.70–1.97	0.571	1.35	0.47–3.86	0.506	1.25	0.65–2.41
	Bridging therapy	0.252	1.51	0.75–3.05	0.252	0.41	0.09–1.90	0.005	3.05	1.39–6.67
	NIHSS	<0.001	1.10	1.05–1.15	0.001	1.09	1.03–1.15	0.916	1.00	0.96–1.05
	Smoking	0.014	0.50	0.29–0.87	0.096	2.59	0.84–7.97	0.644	1.18	0.58–2.39
	Drinking	0.209	1.41	0.82–2.42	0.939	1.04	0.36–3.04	0.002	3.18	1.56–6.51
	AF	0.028	0.41	0.18–0.91	0.124	2.49	0.78–7.93	0.922	1.05	0.42–2.59
	CHD	0.351	0.76	0.42–1.36	0.757	1.19	0.40–3.49	0.017	0.40	0.19–0.85
	Hypertension	0.444	1.22	0.73–2.02	0.963	0.97	0.31–3.02	0.031	2.08	1.07–4.05
	Diabetes	0.768	1.11	0.56–2.20	0.611	0.67	0.14–3.16	0.401	1.44	0.61–3.38
	Prior Stroke	0.959	0.98	0.51–1.90	0.400	0.53	0.12–2.31	0.879	1.06	0.49–2.31
	Dyslipidemia	0.525	1.22	0.66–2.28	0.480	1.52	0.47–4.87	0.230	0.61	0.28–1.36
	sBP	0.344	1.01	0.99–1.02	0.729	1.00	0.98–1.03	0.773	1.00	0.99–1.02
	dBP	0.693	1.00	0.99–1.02	0.255	0.98	0.95–1.01	0.999	1.00	0.98–1.02
	Glucose	0.041	1.11	1.00–1.22	0.130	1.13	0.96–1.34	0.206	1.08	0.96–1.21
	Homocysteine	0.101	1.01	1.00–1.03	0.142	1.02	0.99–1.04	0.362	1.01	0.99–1.03
	Cholesterol	0.949	0.98	0.54–1.77	0.732	1.24	0.37–4.15	0.123	1.86	0.85–4.09
	Triglyceride	0.856	0.97	0.70–1.35	0.330	0.68	0.31–1.48	0.723	0.92	0.59–1.44
	LDL-C	0.968	1.01	0.51–2.00	0.591	1.42	0.39–5.16	0.006	0.28	0.11–0.70
	HDL-C	0.253	1.71	0.68–4.26	0.868	0.86	0.16–4.77	0.336	0.57	0.18–1.80

*Model 1 was adjusted for gender, age, BMI, ONT, dosage of rt-PA, bridging therapy, and NIHSS; Model 2 was adjusted for drinking, smoking, prior stroke, AF, CHD, hypertension, diabetes mellitus, prior stroke, and dyslipidemia; Model 3 was adjusted for sBP, dBP, baseline glucose, homocysteine, cholesterol, triglyceride, LDL-C, and HDL-C; Model 4 was adjusted for Model 1 + Model 2 + Model 3.*

*OR, Odds ratio; 95% CI, 95% confidence interval; BMI, Body mass index; ONT, Onset to needle time; NIHSS, The National Institutes of Health Stroke Scale; AF, Atrial fibrillation; CHD, Coronary heart disease; sBP, Systolic blood pressure; dBP, Diastolic blood pressure; LDL-C, Low density lipoprotein cholesterin; HDL-C, High density lipoprotein cholesterin; NT-proBNP, N-terminal of the prohormone brain natriuretic peptide.*

### Individualized Prediction Model

**FIGURE 3:**
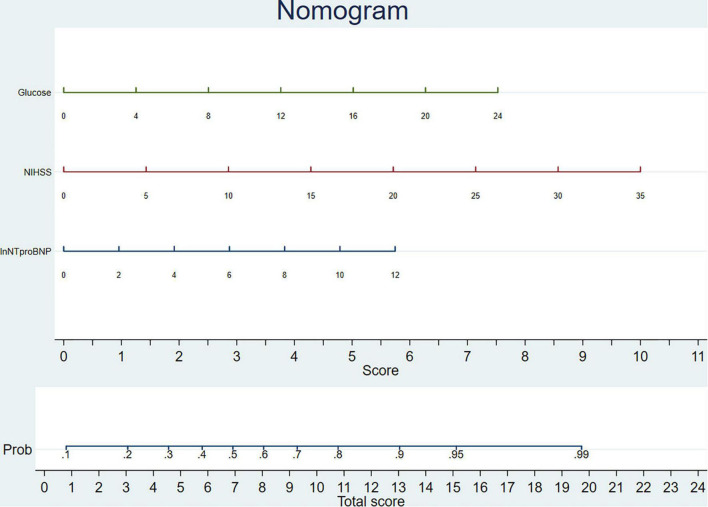
Nomogram of individualized prediction model to predict 3-month poor functional outcome. The nomogram was composed of two areas, the upper area was designed to calculate the total score as the sum of scores for ln(NT-proBNP), National Institutes of Health Stroke Scale (NIHSS), and glucose. The lower area was designed to get the probability of patients to get a poor functional outcome.

**FIGURE 4:**
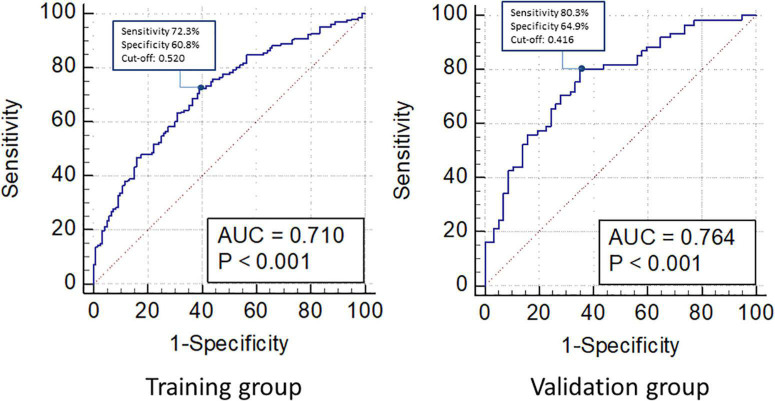
Receiver operating characteristic (ROC) curve for individualized prediction model to predict 3-month poor functional outcome in training and validation group. In the training group, area under the curve (AUC) was 0.710. Cutoff point was marked on the figure with sensitivity 72.3%, specificity 60.8%, and cutoff value was 0.520. In the validation group, AUC was 0.764. Cutoff point was marked on the figure with sensitivity 80.3% and specificity 64.9% and cut off value was 0.416.

**FIGURE 5:**
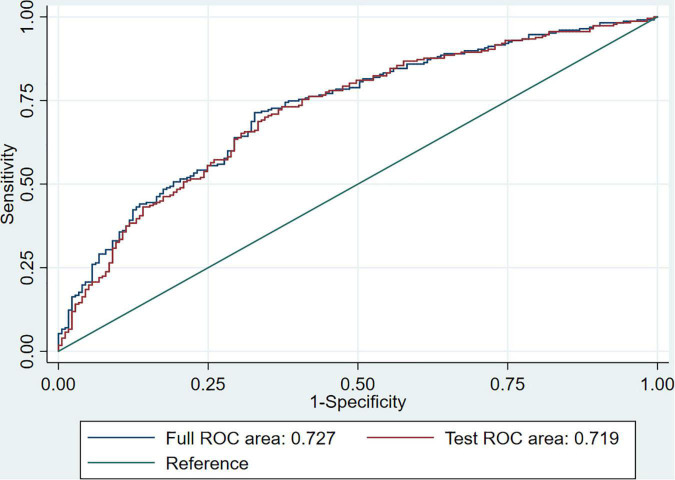
ROC curve for 10-fold cross-validation. The full ROC area was 0.727, and the test ROC area was 0.719 for 10-fold cross-validation.

An individualized prediction model for functional outcomes was established based on the results of the univariate analysis. The prediction model was composed of ln(NT-proBNP), NIHSS scores, and baseline glucose levels. A nomogram was drafted accordingly and is depicted in Figure 3. In the training group, the AUC for the individualized prediction model was 0.710 (95%CI 0.651–0.770), suggesting good discrimination. The *P*-value for the Hosmer-Lemeshow test was 0.998 > 0.05, suggesting good calibration. To test the performance of the individualized prediction model, it was tested using the validation group. The AUC for the model to predict poor functional outcomes was 0.764 (95% CI 0.679–0.850), suggesting good discrimination. The *P*-value for the Hosmer-Lemeshow test was 0.197 >0.05, suggesting good calibration. With all included patients, the full ROC area was 0.727 and the test ROC area was 0.719, which were similar. It suggested good stability of the present model. The ROC curve for the model to predict poor functional outcomes in the training and validation groups and the ROC curve for 10-fold cross-validation are depicted in Figures 4, 5.

## Discussion

The findings of the present study demonstrated that NT-proBNP levels were associated with hemorrhagic transformation in patients who have undergone intravenous thrombolysis, and NT-proBNP levels were associated with poor 3-month functional outcomes and mortality. An individualized prediction model composed of ln(NT-proBNP), NIHSS scores, and baseline glucose levels was then established. Both calibration and discrimination were suitable for the individualized prediction model. Therefore, it is considered feasible to use NT-proBNP levels as a prognostic biomarker in patients with stroke who have undergone intravenous thrombolysis.

N-terminal pro-brain natriuretic peptide is released from the ventricular myocardium in response to stretching (Daniels and Maisel, 2007). Its elevation in patients with stroke, which is associated with poor outcomes, could be explained by stroke-induced cardiac dysfunction. Stroke can activate the hypothalamic-pituitary-adrenal axis, causing elevated sympathetic tone and a catecholamine surge. The over-activation of beta-receptors by a catecholamine surge could cause calcium overload, oxidative stress, and prolonged contraction, leading to cardiomyocyte dysfunction and elevated NT-proBNP levels (Chen et al., 2017; Battaglini et al., 2020). Stroke may also affect the central autonomic network, such as the insular cortex. Previous studies have reported that ischemic lesions of the insular cortex are correlated with blood pressure variations, arrhythmias, and myocytolysis, and insular cortex infarcts have a stronger association with long-term outcomes in patients with stroke (Sander et al., 2001; Ozdemir and Hachinski, 2008; Gopinath and Ayya, 2018). Gut microbiome dysbiosis may also participate in the process of stroke-induced cardiac injury (Chen et al., 2017; Cryan et al., 2020). The association between NT-proBNP levels and poor stroke outcomes could also be explained as concomitant cardiac dysfunction, which is reflected by elevated NT-proBNP levels. Previous studies have reported that NT-proBNP levels are strongly associated with cardioembolic stroke and AF (Llombart et al., 2015; Zhang K. et al., 2021). Cardioembolic stroke tends to be much more severe with a higher risk of mortality and recurrence (Ferro, 2003). Studies have reported that cardioembolic stroke is less reactive to intravenous thrombolysis, which tends to reduce the possibility of early improvement and excellent clinical outcomes, and results in a higher possibility of hemorrhagic transformation, as compared with atherosclerosis-derived stroke (Vaclavik et al., 2018). AF-related stroke was also revealed to have a higher risk of mortality and hemorrhage (Saposnik et al., 2013), and the association between AF and mortality was also reported in the present study. NT-proBNP could also reflect atrial cardiopathy (Kamel et al., 2018), which could be regarded as a precursor to AF and also causes embolic events (Shen et al., 2019). In the present study, the proportion of AF was similar in patients with good and poor functional outcomes, while NT-proBNP levels were significantly elevated in patients with poor outcomes. The association between elevated NT-proBNP levels and poor stroke outcomes might be explained by concomitant atrial cardiopathy, which promotes thrombus formation and causes embolic stroke with a mechanism similar to that of AF (Shen et al., 2019). Some studies also suggested brain secretion of NT-proBNP, and the concentration of NT-proBNP in cerebrospinal fluid could greatly increase after brain injury (Manea et al., 2015; Ru et al., 2021). Stroke could induce blood brain barrier dysfunction and increased permeability (Jiang et al., 2018), and NT-proBNP might pass through blood brain barrier and causes increased blood NT-proBNP levels, reflecting the brain injury. However, the mechanism was less elucidated; the mechanism and whether increased cerebrospinal fluid NT-proBNP is harmful to brain deserve further investigation.

The association between NT-proBNP levels and stroke mortality has been demonstrated. A meta-analysis reported an association between NT-proBNP levels and all-cause mortality (OR 2.43, 95% CI 1.62–3.64) and an association between NT-proBNP levels and cardiovascular mortality (OR 2.01, 95% CI, 1.55–2.61) (Zhao et al., 2020). Considering the NT-proBNP levels in patients who underwent intravenous thrombolysis, one study reported that NT-proBNP levels were associated with 3-month mortality in patients with stroke who underwent intravenous thrombolysis (OR 1.334, 95% CI 1.020–1.745) (Zhang X. et al., 2021). In the present study, NT-proBNP levels were independently associated with 3-month mortality, which is consistent with the findings of the previous study.

The association between NT-proBNP levels and functional outcomes has also been reported in previous studies. A meta-analysis of five studies found that NT-proBNP levels were associated with functional outcomes (OR 1.68, 95% CI 1.13–2.50) (Otaki et al., 2019; Zhao et al., 2020). Considering the performance of NT-proBNP in patients who underwent intravenous thrombolysis, a previous study reported a tendency of poor functional outcomes as NT-proBNP levels increased (Zhang X. et al., 2021). In our study, we found an independent association between NT-proBNP levels and poor 3-month functional outcomes in patients who underwent intravenous thrombolysis.

Another major finding of the present study was the correlation between elevated NT-proBNP levels and hemorrhagic transformation in patients with stroke who underwent intravenous thrombolysis. To the best of our knowledge, this association has not been reported previously. Hemorrhagic transformation is a major complication in patients with stroke who underwent intravenous thrombolysis. Hemorrhagic transformation could impact subsequent antiplatelet treatments; severe symptomatic intracranial hemorrhage could be associated with a mortality rate of up to 50% (National Institute of Neurological Disorders and Stroke rt-Pa Stroke Study Group, 1995; Yaghi et al., 2014). The association between elevated NT-proBNP levels and intracerebral hemorrhage has been demonstrated in previous studies (Di Castelnuovo et al., 2019), and elevated NT-proBNP levels were associated with a larger volume of hematoma and poor outcomes (Li et al., 2017, 2018). Our findings demonstrate that NT-proBNP levels are independently associated with hemorrhagic transformation in patients with stroke who have undergone intravenous thrombolysis. Elevated NT-proBNP levels are closely correlated with cardioembolic stroke or cardiac etiology (Llombart et al., 2015; Zhang K. et al., 2021). Patients with cardioembolic stroke or AF-related stroke had a higher risk of hemorrhagic transformation and poor outcomes (Saposnik et al., 2013; Vaclavik et al., 2018). One potential reason for the elevated NT-proBNP levels in hemorrhagic transformation could be a defined or undefined cardiac etiology of stroke. Another potential reason for elevated NT-proBNP levels in hemorrhagic transformation could be that hemorrhagic transformation aggravated the ischemic stroke-induced neurological injury (Hajdinjak et al., 2012) and could also aggravate stroke-induced cardiac dysfunction in the same way (Chen et al., 2017; Battaglini et al., 2020). However, whether thrombolytic therapy could impact the levels of NT-proBNP is still unknown. Future studies are required to further elucidate the mechanisms underlying elevated NT-proBNP levels in patients with stroke who have undergone intravenous thrombolysis.

N-terminal pro-brain natriuretic peptide was independently associated with mortality, poor functional outcome, and hemorrhagic transformation in patients with stroke who underwent intravenous thrombolysis. However, it became less prominent in subtypes of stroke, which might be explained by unequal division of patients with good or poor outcome into different subtypes of stroke. Just like patients with small artery occlusion tended to have better outcome, while cardioembolism tended to have worse outcome (Ferro, 2003; Ntaios et al., 2016), a greater proportion of poor outcome could be observed in stroke of cardioembolism rather than small artery occlusion. NT-proBNP might achieve its prognostic validity through identifying specific etiologies of stroke, which are accompanied with poor outcome and elevated NT-proBNP, such as cardioembolic stroke or some patients with severe cardiac dysfunction (Llombart et al., 2015; Yaghi et al., 2016; Kamel et al., 2018). The levels of NT-proBNP were diverse in different subtypes of stroke (Iltumur et al., 2006), while they might be less distinct in each subtype. That could be the reason for unsatisfactory prognostic value of NT-proBNP in specific subtypes of stroke. The association between NT-proBNP and functional outcome in stroke with large artery atherosclerosis and undetermined etiology, and association between NT-proBNP and hemorrhagic transformation in stroke with large artery atherosclerosis were observed. Multiple mechanisms might also be behind the prognostic value of NT-proBNP in patients with stroke who underwent intravenous thrombolysis, which could include activated hypothalamic-pituitary-adrenal axis and central autonomic network, impaired blood brain barrier, or unidentified cardiac etiology (Chen et al., 2017; Gopinath and Ayya, 2018; Jiang et al., 2018; Battaglini et al., 2020). The mechanisms behind the prognostic validity of NT-proBNP in patients with stroke who underwent intravenous thrombolysis were not totally elucidated and remained to be illuminated by future studies.

N-terminal pro-brain natriuretic peptide levels have been used in many models to predict mortality after stroke, as reported in previous papers. The combination of NT-proBNP levels and NIHSS scores could identify 89.47% of deceased patients (Chen et al., 2012). The combination of NT-proBNP and troponin T levels could achieve 93% sensitivity and 96% specificity for predicting inhospital mortality of patients with stroke (Hajdinjak et al., 2012). In the present study, we established an individualized prediction model to predict 3-month functional outcomes, which were composed of ln(NT-proBNP), NIHSS scores, and baseline glucose, and a nomogram was drafted accordingly. The nomogram was composed of two areas, the upper area was designed to calculate the total score, and the lower area was designed to get the probability of patients to get a poor functional outcome. The values of ln(NT-proBNP), NIHSS scores, and glucose levels could be marked on the homonymic lines. Vertical lines should then be drawn from the marks to get intersections on the horizontal axis of the upper area, and the scores for ln(NT-proBNP), NIHSS, and glucose levels could be acquired. The total score is obtained by adding the three scores mentioned above, and it is marked on the horizontal axis of the lower area to get the probability for patients to get a poor functional outcome. The individualized prediction model could be used in clinical scenarios with the nomogram. With good discrimination and calibration in both training and validation groups, the model based on NT-proBNP levels appears to be feasible for predicting the 3-month functional outcomes.

There are several limitations in this study. First, the study data were retrospectively analyzed, although prospectively recruited. Second, all the data involved were collected from one hospital. The performance of the individualized prediction model was not tested in other populations, where the performance of the nomogram could be less convincing. Third, NT-proBNP levels were only tested after intravenous thrombolysis and were not performed before thrombolysis or after discharge. We only demonstrated the prognostic validity of NT-proBNP levels but failed to observe the impact of thrombolysis agents on NT-proBNP levels and did not track fluctuations in NT-proBNP levels after discharge. Fourth, stroke with other etiology was not analyzed, and stroke subtypes were not fitted into multivariate analysis and prediction model, because the population for stroke with other etiology was small where bias might derive from. Larger cohort was supposed to be established to observe prognostic value of NT-proBNP in all subtypes of stroke and excavating underlying mechanisms.

## Conclusion

To the best of our knowledge, this is the first report of the association between NT-proBNP level and hemorrhagic transformation in patients with stroke who have undergone intravenous thrombolysis. An association between poor 3-month functional outcomes and mortality was also noted in patients who underwent intravenous thrombolysis. An individualized prediction model, together with a nomogram, to predict the 3-month functional outcome was established. Our findings indicate that NT-proBNP levels could be used as a prognostic biomarker in patients with stroke who have undergone intravenous thrombolysis.

## Data Availability Statement

The raw data supporting the conclusions of this article will be made available by the authors, upon request to the corresponding author.

## Ethics Statement

The studies involving human participants were reviewed and approved by the Ethics Committee of the First Hospital of Jilin University. The patients/participants provided their written informed consent to participate in this study.

## Author Contributions

K-JZ, Z-NG, and YY: conceptualization. K-JZ, HJ, and RX: data curation. PZ: methodology. K-JZ, RX, and PZ: software. K-JZ and HJ: writing–original draft. Z-NG and YY: supervision, and writing–review and editing. All authors contributed to the article and approved the submitted version.

## Conflict of Interest

The authors declare that the research was conducted in the absence of any commercial or financial relationships that could be construed as a potential conflict of interest.

## Publisher’s Note

All claims expressed in this article are solely those of the authors and do not necessarily represent those of their affiliated organizations, or those of the publisher, the editors and the reviewers. Any product that may be evaluated in this article, or claim that may be made by its manufacturer, is not guaranteed or endorsed by the publisher.
